# Interventions for women who report domestic violence during and after pregnancy in low- and middle-income countries: a systematic literature review

**DOI:** 10.1186/s12884-020-2819-0

**Published:** 2020-03-06

**Authors:** Diandra Daley, Mary McCauley, Nynke van den Broek

**Affiliations:** Centre for Maternal and Newborn Health, Liverpool School of Tropical Medicine, Pembroke Place, Liverpool, L3 5QA UK

**Keywords:** Domestic violence, Pregnancy, Postnatal, Interventions, Low resource settings, Low- and middle-income countries

## Abstract

**Background:**

Domestic violence is a leading cause of social morbidity and may increase during and after pregnancy. In high-income countries screening, referral and management interventions are available as part of standard maternity care. Such practice is not routine in low- and middle-income countries (LMIC) where the burden of social morbidity is high.

**Methods:**

We systematically reviewed available evidence describing the types of interventions, and/or the effectiveness of such interventions for women who report domestic violence during and/or after pregnancy, living in LMIC. Published and grey literature describing interventions for, and/or effectiveness of such interventions for women who report domestic violence during and/or after pregnancy, living in LMIC was reviewed. Outcomes assessed were (i) reduction in the frequency and/or severity of domestic violence, and/or (ii) improved physical, psychological and/or social health. Narrative analysis was conducted.

**Results:**

After screening 4818 articles, six studies were identified for inclusion. All included studies assessed women (*n* = 894) during pregnancy. Five studies reported on supportive counselling; one study implemented an intervention consisting of routine screening for domestic violence and supported referrals for women who required this. Two studies evaluated the effectiveness of the interventions on domestic violence with statistically significant decreases in the occurrence of domestic violence following counselling interventions (488 women included). There was a statistically significant increase in family support following counselling in one study (72 women included). There was some evidence of improvement in quality of life, increased use of safety behaviours, improved family and social support, increased access to community resources, increased use of referral services and reduced maternal depression. Overall evidence was of low to moderate quality.

**Conclusions:**

Screening, referral and supportive counselling is likely to benefit women living in LMIC who experience domestic violence. Larger-scale, high-quality research is, however, required to provide further evidence for the effectiveness of interventions. Improved availability with evaluation of interventions that are likely to be effective is necessary to inform policy, programme decisions and resource allocation for maternal healthcare in LMIC.

**Trial registration:**

Systematic review registration number: PROSPERO CRD42018087713.

## Introduction

Domestic violence is a leading yet preventable cause of ill-health, disability and death, affecting one in three women worldwide [1]. Sustainable Development Goal 5 (SDG 5) is to achieve gender equality and empower all women and girls, and sub-target (SGD 5.2) is to eliminate all forms of violence against women, including domestic violence, worldwide by 2030 [2]. Violence against women is defined as ‘any act of gender-based violence that results in, or is likely to result in, physical, sexual or psychological harm or suffering to women, including threats of such acts, coercion or arbitrary deprivation of liberty, whether occurring in public or in private life’ [3]. Domestic violence can first occur and/or increase in frequency and/or severity during and/or after pregnancy [4]. Domestic violence during and after pregnancy can have serious adverse effects on a woman’s physical, psychological and social health and well-being [5–7]. Recent studies estimate that one in three women report domestic violence during and/or after pregnancy, but prevalence varies depending on the setting [5]. Domestic violence is often considered a ‘taboo’ subject and may be unreported or underestimated, especially for women living in low- and middle-income county (LMIC) settings [6, 7].

A current global priority is to ensure that every woman in every setting has access to the highest attainable standard of health and well-being [8]. It is important that healthcare providers are supported and empowered to provide high-quality care to women that extends beyond physical health and includes the screening and effective management of psychological and social well-being [7–9]. In many high-income countries domestic violence is recognised as a public health problem and there are referral systems and interventions available for all women, such as social services support, counselling, psychotherapy, education, and access to aid and refuge centres [10–12]. Pregnancy is a recognised risk factor for domestic violence, and in many high-income countries health policies are in place whereby all women are routinely screened during antenatal care by trained healthcare providers and effective interventions are available for women who need this [9, 13]. In LMIC, pregnancy is often the first time a woman will access healthcare and often there may be ‘missed opportunities’ to provide comprehensive and holistic care for women. Healthcare providers are increasingly becoming aware of the burden of domestic violence women living in LMIC are suffering during and after pregnancy, and there is a move towards routine screening using standardised methods in some settings [7].

## Objective

To review systematically the available evidence describing types of interventions for, and/or effectiveness of such interventions for women living in LMIC, who report domestic violence during and/or after pregnancy.

## Materials and methods

### Data sources and search strategy

This protocol is registered in PROSPERO (CRD42018087713). Relevant articles published up to March 2019, were identified using a structured search strategy in five electronic databases: CINAHL Plus, Global Health, Medline, Web of Science and the Cochrane Library. In addition, grey literature was searched using Google Advanced Search, Google Scholar, Bielefeld Academic Search Engine (BASE), World Bank Open Knowledge Repository and World Health Organization (WHO) Global Health Observatory up to March 2019. A search strategy was developed using thesaurus (including MeSH), and free-text terms for domestic violence, pregnancy, developing countries and associated keywords, were used as main search terms (Supplementary Table 1). Language was limited to English, but no limit was applied to the publication year. Reference lists and bibliographies of key topic articles were also searched to identify any additional relevant articles.

### Inclusion and exclusion criteria

All studies reporting on domestic violence in women during pregnancy, childbirth or up to 12 months postnatal in LMIC, and, (a) included assessment or severity of domestic violence; (b) described and/or evaluated any type of screening, referral or intervention for domestic violence were included. Publications were examined to ensure that they did not feature the same data set as that presented in other articles. For assessment of effectiveness of interventions, outcomes were identified at the start of the review. Outcomes assessed included (i) a reduction of the frequency and/or severity of domestic violence; and/or (ii) physical (pregnancy and/or maternal health outcomes; neonatal health outcomes); psychological (depression, anxiety, stress, post-traumatic stress disorder); and/or social health measures (quality of life, help-seeking and safety behaviours, perceived family and/or social support; access to community resources; use of referral services). There was no limitation to the type of study design used.

### Selection and data extraction

Two researchers conducted screening of titles and abstracts and evaluation of full-text papers independently with reasons for exclusion recorded and discrepancies discussed with a third researcher. Information from included papers was extracted into a pre-designed summary table and included data on location of study, study dates, study design, study population, types of screening, referral and/or intervention identified, methods of evaluation and the timing (pregnancy phase) of the assessment (Table 1). Throughout the reviewing and extraction processes, articles where uncertainty existed were discussed with a fourth researcher and consensus reached.

**Table 1:** Summary table of included studies

No.	Author, year	Study design	Study participants	Data collection tools used	Type of intervention	Effect of intervention on domestic violence	Effect of intervention on health
1.	Cripe 2010	Randomised controlled trial (two-arm trial with individual randomisation)	220 pregnant women attending antenatal care; aged 18–45 years old, between 12- and 26-weeks’ gestation. 110 women were randomly assigned to each intervention arm.	Modified Abuse Assessment Screen; Short Form Health Survey (SF-36); Modified Safety Behaviour Checklist; Modified community resource use assessment.	Counselling Standard intervention – details of organisations providing support Empowerment intervention – 30 min interview.	Not assessed.	Women had higher scores for physical functioning, physical and emotional scales, vitality, and social functioning at post-intervention interview. Increase in number of women who adopted safety behaviours in intervention arm (1.8 to 30.3%). Women were more likely to seek help from community resources, particularly from the church and the police.
2.	Matseke 2013	Pre/post-intervention	160 pregnant women attending primary healthcare clinics for HIV post-test counselling aged 18 years or older. 160 women were assessed pre-intervention; 82 women followed up post-intervention (52.5% retention rate).	Authors own screening form; Danger Assessment Scale (20 item questionnaire).	Counselling Twenty minute one-to-one intervention and 3 months follow-up.	The pre-intervention mean danger assessment score declined significantly from 6.0 to 2.8 after 3 months (*p* < 0.001).	Not assessed.
3.	Turan 2013	Mixed methods evaluation: Cross-sectional study focus group and in-depth interviews	134 pregnant women attending antenatal care, post-intervention evaluation: Clinic staff and community volunteers (two focus groups; *n* = 17, male and female)	Anonymous risk assessment form; focus group discussions and in-depth interviews using own topic guides.	Routine screening and referral programme.	Not assessed.	Community awareness on domestic violence increased; community collaboration helped to find local solutions for victims, particularly in rural and low-resource settings. The intervention aided pregnant women in accessing domestic violence services; particularly for rural women who had less access to services. 53% of women reporting violence accepted referrals to local support resources.
4.	Krishnan 2012	Qualitative: focus group discussions and in-depth interviews	Two study groups: 20 pregnant women attending antenatal care or in the local community, aged 18 to 30 years; and 20 mothers-in-law participants	Focus group discussions and in-depth interviews using own topic guides.	Counselling Two 3-h sessions with daughters-in-law Five 3-h sessions with mothers-in-law One joint 3-h with both.	Not assessed.	Daughter-in-laws (pregnant participants) reported an increase in family support, as relationships with their mothers-in-law had improved.
5.	Mutisya 2018	Quasi-experiment	288 pregnant women attending antenatal care alone; aged 18–45 years old; in the first or second trimester of pregnancy. 144 women were randomly assigned to each intervention arm.	Abuse Assessment Screen; a modified pregnancy version of the Composite Abuse Scale; Edinburgh Postnatal Depression Scale.	Counselling A minimum of three 30–35 min sessions over four months.	After adjusting for baseline scores, the differences in violence and physical violence scores between the intervention and control group were significant (*p* < 0.001), with small effect sizes (0.196 and 0.305, respectively).	After adjusting for baseline scores, the intervention group had significantly lower mean antepartum depression scores (measured as EDPS ≥13) compared to the usual care post-intervention, F (1,280) = 106.25, *p* < 0.001, with a medium between the groups with an effect size of 0.500.
6.	Akor 2019	Single-blinded randomised controlled trial (two-arm trial with individual randomisation)	72 pregnant women attending for antenatal care; most women aged 20–34 years old (86%), less than 34 weeks gestation. 36 women were randomly assigned to each intervention arm.	Abuse Assessment Scale; Systematic clinical outcome and routine evaluation, SCORE-15.	Counselling Three counselling sessions at two-weekly intervals; incorporated into routine antenatal visits.	Not assessed.	Family function was assessed across three dimensions: family communication, family support and family difficulty. Women in the intervention group had an improved mean family function score (2.92 0.92 to 2.16 0.63; this improvement was statistically significant (*p* < 0.0001). Women in the control group had an improvement in mean family score, but this change was not statistically significant (*p* < 0.116).

### Quality assessment

The risk of bias and the methodological quality of included studies was assessed using the Grading of Recommendations, Assessment, Development and Evaluation (GRADE) tool by one researcher and checked by a second researcher. Using this approach, studies were graded as high, moderate, low or very low quality. Once an a-priori ranking of evidence was assigned based on study design, studies could be downgraded for five additional reasons: risk of bias, inconsistency, indirectness, imprecision, and publication bias. The grading of the quality of evidence and reasons for downgrading the quality of evidence was documented in a pre-designed summary table (Supplementary Table 2).

### Data synthesis

A narrative synthesis approach was used to describe outcomes including: prevalence of the domestic violence; descriptions of interventions; effect of intervention on the frequency or severity of domestic violence; and the effect of the intervention on physical (pregnancy and/or maternal health outcomes; neonatal health outcomes); psychological (depression, anxiety, stress, post-traumatic stress disorder); and/or social health outcomes (quality of life, help-seeking and safety behaviours, perceived family and/or social support; access to community resources; use of referral services). Where a standardised data collection tool was used, this was described. The methodology and results of studies belonging to the same outcome category were compared for similarities and differences.

## Results

**Fig. 1:**
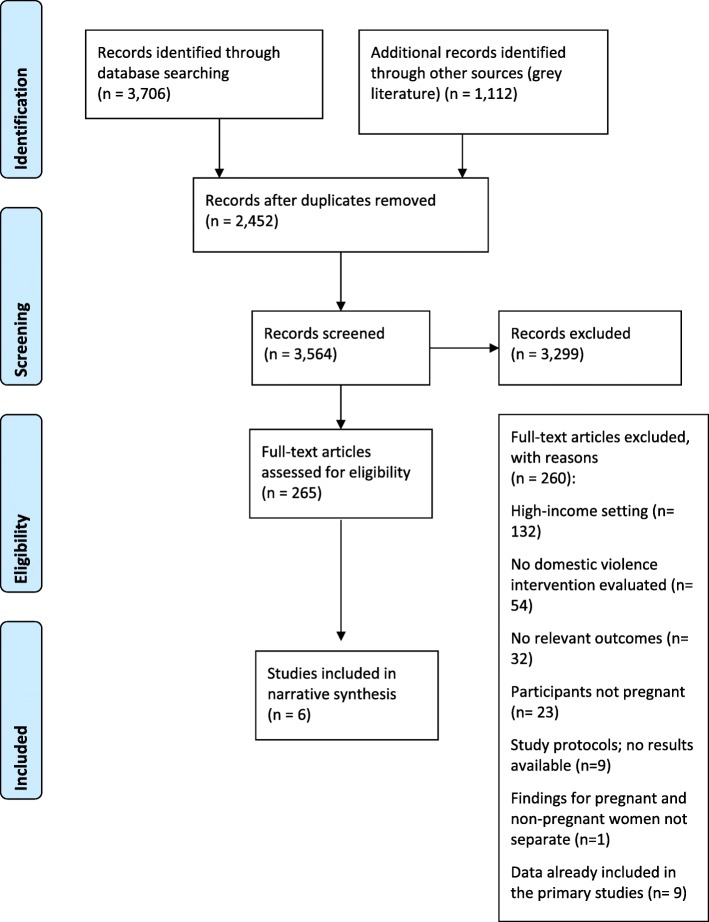
PRISMA flow diagram

By combining the search terms, 4818 articles were identified, and after screening for relevance, 265 were retrieved for full text review (Fig. 1). Upon applying the eligibility criteria, six studies were included in the review. These articles were considered key papers and their reference lists were examined to identify any additional relevant articles. However, no new articles were identified.

### Characteristics of included studies

Six studies were published between 2010 and 2019, assessing 894 pregnant women. Table 1 provides an overview of the types of interventions and outcomes evaluated across included studies.

### Study design and interventions

A variety of study designs were used to implement and evaluate interventions. One mixed method study evaluated a large-scale community programme involving screening for domestic violence among pregnant women and referral for those requiring this [14]. Two studies were randomised controlled trials that compared the intervention of counselling to routine care [15, 16]. One quasi-experiment study [17] compared the intervention to normal routine antenatal care in that setting. However, due to ethical considerations, this was supplemented by a referral card listing local resources providing help or advice for domestic violence in case women wished to access this [17]. A pre-post intervention study design [18] was used to evaluate the effect of a supportive counselling. Qualitative interviews and focus group discussions were used to explore the effect of counselling on mother and daughter-in-law relationships [19].

### Study settings

All studies were conducted in middle-income countries: two in Kenya [14, 17]; and one each in Peru, Nigeria, South Africa and India. [15, 16, 18, 19]. Two studies were conducted at secondary healthcare facility level [15, 16] and four were conducted in primary health care facilities [14, 17–19].

### Study population

All studies recruited pregnant women. Four studies recruited women during antenatal care visits [14–17]. One study recruited women from antenatal care visits and via community outreach [19]. One study recruited women as they attended a healthcare facility for HIV post-test counselling [18]. The age range, gestation, parity, socioeconomic status and social history of participants were reported inconsistently across included studies. No studies recruited pregnant adolescents or women after childbirth. Sample sizes ranged from 20 to 288 pregnant women per study.

### Screening and referral

One study offered routine screening and referral and reported that 53% of pregnant women who reported domestic violence, accepted referral to local support resources [14]. Healthcare providers noted that during the screening and referral intervention, community awareness regarding domestic violence increased [14]. There were observed benefits of screening, particularly for women living in a rural area where community collaboration helped to facilitate referrals and to find local solutions for women living in these areas [14]. However, socioeconomically vulnerable women were often reluctant to take legal action for fear of ‘breaking the family’ and preferred to ‘solve things at home’ [14].

### Effect of interventions on domestic violence

In the largest included study of 288 women, women who received psychosocial counselling as well as those in the comparison group showed a reduction in the frequency and severity of domestic violence, and this included both physical and emotional abuse [17]. However, this reduction was much higher in the intervention group. Differences in scores for domestic violence and physical violence between the groups were statistically significant with small but not negligible effect sizes (difference in scores 0.196 and 0.305 respectively). Although the effects of the intervention on severe combined violence, emotional abuse and harassment were statistically significant, the effects were small (0.046, 0.078 and 0.086 respectively) [17]. In another study, there was a 4% decrease in the self-reported frequency and severity of physical violence; 13.5% decrease in sexual abuse, and 27% decrease in physical abuse reported by women who received an empowerment intervention [18]. The pre-intervention mean assessment score declined significantly from 6.0 to 2.8 following the intervention and this difference was statistically significant (*p* < 0.001) [18].

### Effect of interventions on women’s health

Mutisya et al. reported a decline in the mean antepartum depression scores using the Edinburgh Postnatal Depression Score from 15.58 (SD 3.74) in the comparison routine care group versus 14.07 (SD 4.27) in the intervention group [17]. However, the intervention group had a significantly lower mean depression score with an effect size of 0.500. Cripe et al. report that women who received counselling scored higher quality of life measures following the intervention [15]. Compared to the comparison group, women who received counselling were more likely to (a) have an emergency money fund (44.6% vs 34.3%), (b) have a safety code with family or friends (19.6% vs 16.2%), (c) ask neighbours to call police if they suspected violence (6.9% vs 1.0%), and (d) have an emergency bag packed (9.0% vs 3.1%). These women were also more likely to seek help from community resources, such as the church and police. However, there were no statistically significant differences for each of these outcomes. Krishnan et al. reported that daughters-in-law (pregnant women) felt more knowledgeable and confident about their health and reported improvements in their communication and coping skills following a counselling intervention during pregnancy [19]. This included talking more openly at home and being able to negotiate during conflicts with their husbands. They also reported increased family support, as relationships with their mothers-in-law had improved [19]. In this study, mothers-in-law also reported greater improvements in their relationships with their daughters-in-law as a result of better communication. Mothers-in-law reported greater recognition of the leadership role in preventing violence, promoting health, and intervening with their sons during domestic conflicts [19]. Akor et al. reported an improvement in family function scores in both the intervention and comparison group. However, only the intervention group had a statistically significantly improvement in mean family function score (*p* < 0.0001) [16].

### Quality of studies

Supplementary Table 2 details the results of an assessment of the quality of evidence for each outcome. Outcome data for perceived family or social support was graded low quality, due to indirectness in intervention and comparison groups, high risk of bias in the contributing study data, study design limitations and small sample sizes. Outcome data for the frequency and/or severity of violence, maternal depression and use of referral services were graded low quality. Reasons for downgrading the evidence include study design limitations and small sample sizes. Outcome data for quality of life, help seeking and safety behaviours, and access to community resources was of moderate quality, largely due to the high-quality study design and low risk of bias for contributing study data. The risk of bias across studies was either unclear or of serious concern. Three of the included studies applied randomisation techniques within their study designs [15–17]. However, it was unclear whether methods were undertaken to conceal the allocation sequence. Intrinsic to the ethical challenges associated with this subject, sample sizes were small (compromising the representativeness of findings) and interventions did not allow for blinding of participants or the personnel providing care. Consequently, the validity of the findings of included studies was compromised; particularly as a lack of blinding may have invited response bias for the primary outcome of this review. Where it had been reported, loss of follow-up was not a major concern for researchers as interventions and data collection had been implemented as part of routine antenatal care. Healthcare providers therefore had enough time to collect outcome data during this time. Retention rates were high for most studies, ranging from 77.3 to 100%. However, attrition bias was a serious concern for one of the included studies, where there was a considerable loss to follow-up (52.5%) [18]. This is likely to reflect the realities and challenges of conducting research exploring this potentially sensitive topic (domestic violence) in a vulnerable population (pregnant women).

## Discussion

### Statement of principal findings

Our study highlights that there are currently very few studies that describe the availability of, and evaluate interventions for, women who report domestic violence during and after pregnancy in LMIC. Five studies examined supportive counselling; and one study implemented an intervention consisting of routine screening for domestic violence and supported referrals for women who required it. Two of the five included studies evaluated the effectiveness of the interventions following implementation [14, 19]. There were behaviour changes as a result of a screening and referral intervention programme for victims of domestic violence, but these measures of change were not statistically significant. Two out of four studies evaluated different forms of supportive counselling and reported a statistically significant decrease in the frequency and severity of domestic violence, particularly physical and sexual abuse [16, 17]. One study reported a statistically significant increase in family functioning following supportive counselling [16]. There is also evidence of improved quality of life, increased use of safety behaviours, greater use of community resources and reduced maternal depression amongst women who received supportive counselling. However, these differences were not statistically significant (Table 1).

### Strengths of the study

To the best of our knowledge, this is one of the first systematic reviews to describe the types and effectiveness of interventions available, for women who report domestic violence during and after pregnancy living in LMIC. This review provides insights into how domestic violence against women during and after pregnancy could be assessed and managed in LMIC settings. This study is also the first review evaluating interventions for violence in pregnancy that critically reviews both quantitative and qualitative data.

### Limitations of the study

The review was limited to studies from LMIC as the burden of domestic violence is expected to be highest in women living in resource poor settings. There was a lack of consistency in the outcomes reported across included studies, as well as a lack of information regarding who provided what type of counselling and what training they had received. In instances where studies did report similar outcomes, we note that researchers used different data collection tools at different points in time. Such heterogeneity made meta-analysis an unsuitable method for synthesising the findings of this review, subsequently limiting the ability to draw sound conclusions about the overall effect of interventions. Furthermore, due to the disparate way in which outcomes were reported, it was not possible to conclusively highlight one intervention that works better than others. None of the included studies investigated maternal health or pregnancy outcomes, such as miscarriage, antepartum haemorrhage, maternal injury or trauma. Similarly, no studies investigated outcomes related to neonatal health; and no studies measured the effect of interventions on maternal stress, anxiety or depression. This review was therefore unable to comment on the effect of interventions on these outcomes. Although the early antenatal stage is considered an important time for screening for domestic violence, domestic violence may first occur or be exacerbated during pregnancy [4]. This systematic review found no studies which included women after childbirth. Thus the existing evidence lacks a holistic evaluation of the potential impact of screening and interventions for domestic violence in women living in LMIC at all stages of pregnancy and after childbirth. All included studies were conducted in countries classified as middle-income by the World Bank; there is lack of evidence from low-income countries and these countries are not represented within this review. The applicability of this evidence to low-income countries are therefore limited, and it is not clear whether such interventions are available and/or effective in such settings. This review was limited to English language.

### How does this study relate to other literature?

The objectives and findings of this review are broadly in agreement with those of a recent systematic review [20], which aimed to identify and assess the effectiveness of interventions currently available for women living in LMIC who report domestic violence. The limited quantity of data found across both systematic reviews reflects the observation that screening and interventions for domestic violence in LMIC is not often part of routine antenatal and postnatal care; a recognition of the importance of introducing such interventions is just starting to emerge, and uptake is relatively slow. Our systematic review also has similar findings to a review that examined the effectiveness of domestic violence interventions on a range of physical, psychological and social health outcomes across different settings [21]. However, most of the evidence in the review by van Parys et al. was from studies conducted in high income countries. Evidence for domestic violence interventions outside of the context of pregnancy is deficient and inconclusive [22, 23]. A number of studies have evaluated the effectiveness of home visitation programmes and the provision of a wallet-sized card listing community resources to reduce violence in pregnancy [24–28]. However, these studies were similarly only conducted in high income countries, limiting the generalisability of the findings to LMIC settings. The WHO have produced clinical and policy guidelines on how to respond to pregnant women who report domestic violence including identification, safety assessment and planning, communication and clinical skills, documentation and provision of referral pathways [29]. However, the feasibility of implementation and acceptability of this guidance in LMIC is currently uncertain [29]. There is emerging evidence that women have a high burden of social morbidity (in addition to physical and psychological ill-health) during and after pregnancy, which to date, has largely been ‘hidden’ and/or underestimated [5]. At present, when women attend for antenatal and postnatal care in LMIC, screening for social morbidity is not routinely available. There is also very limited information regarding how, when and with whom women would like to discuss the issue of domestic violence as part of integrated care during and after pregnancy in LMIC settings, and this requires further exploration.

## Conclusion

This systematic review highlights interventions available for women who experience domestic violence during and after pregnancy in LMIC, including screening, referral and supportive counselling; but high-quality studies to provide evidence for the effectiveness of such interventions are currently lacking. However, there is some evidence that screening and referral for supportive counselling, may reduce the frequency of domestic violence, reduce maternal depression, increase social support, improve family functioning and improve the quality of life for these women. High-quality large-scale studies (that incorporate mixed methods and permit meta-analysis) are required. It would be beneficial to conduct implementation studies including where possible population-based randomised controlled trials to assess the acceptability, feasibility and effectiveness of interventions designed to support women who report domestic violence during pregnancy and after childbirth. In the interim, whilst it may not be feasible and acceptable to apply the same approach across all LMIC, having standardised, internationally agreed guidelines and a screening and management protocol for use as part of integrated antenatal and postnatal care which could be adapted by countries, would ensure that domestic violence is appropriately prioritised as a public health concern and does not remain ‘hidden’. Future research will inform policy-making and contribute to local, national and international initiatives to alleviate domestic violence during and after pregnancy.

## Supplementary information

**Additional file1: Table S1.** MeSH terms and keywords used in the search.

**Additional file 2: Table S2.** GRADE assessment of quality of evidence.

**Additional file 3: Table S3.** PRISMA checklist.

## Data Availability

All the sources of data are publicly available and referenced in the document.

## Abbreviations

BASE: Bielefeld Academic Search Engine
EPDS: Edinburgh Postnatal Depression Score
GRADE: Grading of Recommendations, Assessment, Development and Evaluation
HIV: Human Immunodeficiency Virus
LMIC: Low- and middle-income countries
MeSH: Medical Subject Headings
PRISMA: Preferred Reporting Items for Systematic Reviews and Meta-Analyses
SDG: Sustainable Development Goals
WHO: World Health Organization
