# Supplementary material for: Interventions for women who report domestic violence during and after pregnancy in low- and middle-income countries: a systematic literature review
Source: BMC Pregnancy Childbirth. 2020 Mar 6;20:141. doi: 10.1186/s12884-020-2819-0 (PMC7059681; doi:10.1186/s12884-020-2819-0)
Supplement: Supplementary file 1 — Additional file1: Table S1. MeSH terms and keywords used in the search. [file 12884_2020_2819_MOESM1_ESM.docx]

## Supplementary Table 1: Strategy used to search articles with strings presented for the MEDLINE database

| MEDLINE search strategy and preliminary search results (searched 25^th^ May 2019)  (MH = MeSH heading) | | |
| --- | --- | --- |
| Search number | Search terms | Identified results |
| 1 | (MH “Domestic Violence+”) OR (MH “Intimate Partner Violence+”) | 41, 562 |
| 2 | (MH “Spouse Abuse”) | 7,106 |
| 3 | (MH “Battered Women”) | 2,535 |
| 4 | “intimate partner violence” OR “intimate partner abuse” OR “spous* abuse” OR “battered women” OR “domestic violence” OR “domestic abuse” OR violen* OR “emotional abuse” OR “psychological abuse” OR “sexual abuse” | 79,163 |
| 5 | S1 OR S2 OR S3 OR S4 | 95,349 |
| 6 | (MH “Pregnancy+”) | 832,477 |
| 7 | pregnan* OR “pregnant women” OR “expectant mother*” OR “expectant women” OR postpartum OR postnatal OR “childbearing” OR mother* | 1,127,551 |
| 8 | (MH “Postpartum Period+”) | 58,236 |
| 9 | (MH “Prenatal Care”) | 24,203 |
| 10 | prenatal OR antenatal OR antepartum | 178,789 |
| 11 | S6 OR S7 OR S8 OR S9 OR S10 | 1,175,229 |
| 12 | (MH “Developing Countries”) | 69,901 |
| 13 | “developing countr*” OR “low income” OR “low resource” OR “middle income” OR LMIC OR LIC OR “less developed” OR “underdeveloped” OR “resource poor” OR “poor countr*” OR “sub-Saharan africa” OR “latin america” | 184,989 |
| 14 | asia OR caribbean OR “west indies” OR “south america” OR “central america” OR “global south” | 126,793 |
| 15 | S12 OR S13 OR S14 | 284,024 |
| 16 | S5 AND S11 AND S15 | 877 |
