# Supplementary material for: Interventions for women who report domestic violence during and after pregnancy in low- and middle-income countries: a systematic literature review
Source: BMC Pregnancy Childbirth. 2020 Mar 6;20:141. doi: 10.1186/s12884-020-2819-0 (PMC7059681; doi:10.1186/s12884-020-2819-0)
Supplement: Supplementary file 2 — Additional file 2: Table S2. GRADE assessment of quality of evidence. [file 12884_2020_2819_MOESM2_ESM.docx]

## Suppementary Table 2: GRADE assessment of quality of evidence

| Any intervention to prevent violence versus standard care for preventing or reducing domestic violence against pregnant and/or postpartum women | | | |
| --- | --- | --- | --- |
| **Population:** all pregnant women  **Settings:** countries classified as middle-income by the World Bank (Kenya South Africa, Peru and India)  **Intervention:** any intervention to prevent or reduce violence versus standard antenatal care | | | |
| Outcomes | No. of participants (studies) | Quality of evidence (GRADE) | Comments |
| Frequency and/or severity of domestic violence (physical, sexual and/or psychological) | 448  (2 studies) | ⊕⊕⊝⊝  low^1^ |  |
| Maternal/obstetric health | (0 studies) | See comment | No study reported results for this outcome |
| Neonatal health | (0 studies) | See comment | No study reported results for this outcome |
| Maternal depression | 288  (1 study) | ⊕⊕⊝⊝  low^1^ |  |
| Anxiety | (0 studies) | See comment | No study reported results for this outcome |
| Stress | (0 studies) | See comment | No study reported results for this outcome |
| Post-traumatic stress disorder | (0 studies) | See comment | No study reported results for this outcome |
| Quality of life | 220  (1 study) | ⊕⊕⊕⊝  moderate^2^ |  |
| Help-seeking and/or safety behaviours | 354  (2 studies) | ⊕⊕⊕⊝  moderate^1^ |  |
| Perceived family and/or social support and/or functioning | 174  (3 studies) | ⊕⊕⊝⊝  low^3^ |  |
| Access to community resources | 354  (2 studies) | ⊕⊕⊕⊝  moderate^1^ |  |
| Use of referral services | 134  (1 study) | ⊕⊕⊝⊝  low^3^ |  |
| **GRADE Working Group grades of evidence**  **High quality:** We are very confident that the true effect lies close to that of the estimate of the effect **Moderate quality:** We are moderately confident in the effect estimate: The true effect is likely to be close to the estimate of the effect, but there is a possibility that it is substantially different **Low quality:** Our confidence in the effect estimate is limited: The true effect may be substantially different from the estimate of the effect **Very low quality:** We have very little confidence in the effect estimate: The true effect is likely to be substantially different from the estimate of effect | | | |
| ^1^All studies contributing data had design limitations and small sample size  ^2^Estimate based on small sample size  ^3^All studies contributing data had design limitations, indirectness and small sample size | | | |
